# Comment on “Validity and Reliability of the Iranian Version of the Short Form Social Well Being Scale in a General Urban Population”

**Published:** 2020-04

**Authors:** Mehrdad AMIR-BEHGHADAMI, Masoumeh GHOLIZADEH, Ali JANATI

**Affiliations:** 1. Iranian Center of Excellence in Health Management (IceHM), Department of Health Service Management, School of Management and Medical Informatics, Tabriz University of Medical Sciences, Tabriz, Iran; 2. Student Research Committee, Tabriz University of Medical Sciences, Tabriz, Iran; 3. Tabriz Health Services Management Research Center, Health Management and Safety Promotion Research Institute, Tabriz University of Medical Sciences, Tabriz, Iran

## Dear Editor-in-Chief

We recently read with interest the paper written by Shayeghian, et al (1). First of all, we want to sincerely thank the editors of Iranian Journal of Public Health who have been trying to publish this important article. In addition, we want to extend our gratitude to the authors of this article.

The aim of this letter is to highlight the importance of measuring the content validity of scales in psychometric studies. Content validity as an important component of psychometric properties must be performed independently of the translation process (2). This is assessed using panel of experts by means of qualitative and quantitative approaches (3). This panel must include specialists who have research experience or have worked in the field. In the qualitative approach, experts are requested to assess each item qualitatively in terms of grammar, order of words, using correct and appropriate words and scoring. In order to assess the quantitative content validity by the experts, they are wanted to rank the items using 4-point Likert scales in terms of item each relevance and clarity. Based on the experts scoring, the CVI and modified kappa coefficient are calculated (2, 4). Multi-rater Kappa coefficient has been introduced by literature, which is adjusted for odds agreement (2).

To compute modified kappa coefficient, the probability of chance agreement is obtained for each item by the following formula:

PC=[N!/A!(N−A)!]*.5N.

In this formula, N stands for the number of experts and, A stands for the number of agreeing specialists.

After calculating I-CVI for all the instrument items, finally, kappa is computed by entering the numerical values of probability of chance agreement (PC) and content validity index of each item (I-CVI) in the following formula:

K=(I−CVI−PC)/(1−PC).

The purpose of evaluating the content validity of translated instrument is whether its contents are capable of measuring the specified goals, because only acceptable reliability of the instrument cannot determine its validity (5, 6). Hence, the content validity is assessed before running EFA and determining the dimensions. Based on the literature, items with modified kappa score more than 0.78 showed sufficient agreements. Valid and reliable instruments are developed during psychometric researches, and since these instruments can be used in health-related research researches, then it is essential to consider content validity.
